# Mechanisms of exercise in the treatment of lung cancer – a mini-review

**DOI:** 10.3389/fimmu.2023.1244764

**Published:** 2023-08-24

**Authors:** Zhiwen Luo, Renwen Wan, Shan Liu, Xinting Feng, Zhen Peng, Qing Wang, Shiyi Chen, Xiliang Shang

**Affiliations:** ^1^ Department of Sports Medicine, Huashan Hospital, Fudan University, Shanghai, China; ^2^ Department of Endocrinology, Huashan Hospital, Fudan University, Shanghai, China; ^3^ Department of Sports Medicine, Shanghai General Hospital, Shanghai, China; ^4^ Department of Orthopaedics, Kunshan Hospital of Traditional Chinese Medicine, Kunshan, Jiangsu, China

**Keywords:** lung cancer, exercise, prevention, treatment, anticancer mechanisms, tumor microenvironment

## Abstract

Lung cancer constitutes a formidable menace to global health and well-being, as its incidence and mortality rate escalate at an alarming pace. In recent years, research has indicated that exercise has potential roles in both the prevention and treatment of lung cancer. However, the exact mechanism of the coordinating effect of exercise on lung cancer treatment is unclear, limiting the use of exercise in clinical practice. The purpose of this review is to explore the mechanisms through which exercise exerts its anticancer effects against lung cancer. This review will analyze the biological basis of exercise’s anticancer effects on lung cancer, with a focus on aspects such as the tumor microenvironment, matrix regulation, apoptosis and angiogenesis. Finally, we will discuss future research directions and potential clinical applications.

## Introduction

Lung cancer constitutes a formidable menace to global health and well-being, as its incidence and mortality rates escalate at an alarming pace (1). According to the worldwide cancer data of 2020, lung cancer ranks among the most prevalent malignancies and exhibits the highest mortality rate among all major cancers (2, 3). Annually, more than 2.1 million new cases of lung cancer and approximately 1.8 million deaths are recorded (2). Non-small cell lung cancer (NSCLC) is the most prevalent subtype of lung cancer, accounting for approximately 80% of newly diagnosed cases (4). The 5-year survival rate for this malignancy is a mere 15%, attributable in part to the inadequacy of early detection techniques and the absence of effective treatments for advanced disease (1, 4). Approximately fifty percent of non-small cell lung malignancies contain mutations in the tumor suppressor gene p53, which plays a key role in numerous biological pathways, including DNA repair, cell cycle arrest, apoptosis, senescence, autophagy, and metabolism (5). Currently, treatment modalities entail surgical resection, chemotherapy, radiotherapy, targeted therapy, and immunotherapy (6). Therapeutic options for lung cancer patients hinge upon the cancer type, disease stage, and patient functional status. These interventions may also induce adverse effects, which, in conjunction with the cancer symptoms, impose a considerable burden on patients and exacerbate numerous patient-related outcomes, including exercise capacity and physical function (1). Consequently, it is of utmost importance to seek treatment strategies for lung cancer that are less harmful.

Physical activity serves a vital role in the prevention and management of lung cancer (7). Regular physical activity yields substantial health advantages, mitigating the risk of various chronic health disorders, such as obesity, brain disorders, cardiovascular diseases, and diabetes (8–12). The World Health Organization advises adults to partake in a minimum of 150 minutes of moderate-intensity aerobic exercise weekly, fostering cardiorespiratory, muscular, and skeletal health while diminishing the risk of depression. Exercise correlates with a lower risk of diverse cancers, encompassing colorectal, breast, esophageal, pancreatic, endometrial, and ovarian malignancies (10). Moreover, habitual exercise markedly curtails cancer-related mortality rates following breast and colorectal cancer diagnoses (13, 14). However, the molecular mechanisms underlying exercise’s function in lung cancer prevention and treatment remain obscure.

In the realm of lung cancer therapy, exercise has unequivocally exhibited its efficacy in bolstering the quality of life for patients subjected to arduous treatment modalities (6). A salubrious lifestyle characterised by regular exercise and physical activity is associated with a lower incidence of cancer (including lung cancer) and cancer mortality (10, 15, 16). Exercise also improves the overall physical condition of cancer patients undergoing chemotherapy or surgery, thereby reducing treatment-related adverse effects and complications (6, 7, 17). Accumulating evidence underscores the safety and effectiveness of exercise interventions, with pre-operative exercise considerably decreasing the prevalence of post-operative complications (7). In recent scrutinies concerning individuals afflicted with non-small cell lung cancer, a compelling revelation has surfaced, elucidating the transformative potential of physical exercise. This enlightening research has unveiled significant advancements in terms of ambulatory stamina, maximal exercise capability, breathlessness alleviation, reduction in hospitalization risk, and amelioration of postoperative pulmonary complications (2, 3, 18).

Exercise constitutes an essential component in both the prevention and management of lung cancer, with potential underlying biological mechanisms comprising p53-mediated apoptosis, inhibition of lung cancer cell proliferation and survival, augmentation of host immunity, facilitation of immune cell infiltration, refinement of the tumor microenvironment, attenuation of chronic inflammation, activation of DNA repair enzymes, and fortification against oxidative stress (3, 5, 7, 18–24). Furthermore, research has indicated that aerobic exercise and high-intensity interval training exert therapeutic effects on tumors (18, 22, 25). By modulating the microenvironment of tumors and boosting the activity of immune cells such as T lymphocytes and NK cells, exercise can boost immune function and aid in the fight against tumors (3, 17–20).

Although further investigation of the potential anti-tumor effects of combining exercise with immunotherapy is warranted, the implementation of exercise in lung cancer treatment has already shown promise (3, 18). This study chiefly synthesizes all recent fundamental research on exercise’s anti-tumor effects, offering an exhaustive overview of established mechanisms, with the intent of aiding scholars in their investigation of exercise’s anti-cancer properties and furnishing a theoretical foundation for clinical trials.

### The influence of exercise on the tumor immune microenvironment (TIME) of lung cancer

Within the Tumor Microenvironment (TME), three distinct classifications have been identified, namely “Immune inflamed,” “Immune desert,” and “Immune excluded,” based on the infiltration and distribution patterns of immune cells (26–30). In an ‘Immune inflamed’ TME, immune cells are widely infiltrated within the tumor tissues, while in an ‘Immune desert’ TME, immune cells are virtually absent within and around the tumor. An ‘Immune excluded’ TME sees immune cells primarily concentrated at the tumor margins (26, 27, 30).

An increasing body of research suggests that exercise may facilitate the shift in the tumor microenvironment class (13, 31). Although the precise mechanisms are still under investigation, one possible explanation is that exercise may modulate both systemic and local immune responses (19, 22). For instance, exercise may enhance systemic immune responses by augmenting the number of immune cells in circulation; simultaneously, it may influence the activities of immune cells within the tumor by adjusting the local environmental conditions surrounding the tumor, such as oxygen and nutrient supply, as well as the extent of inflammatory and stress responses (31, 32). This could lead to the transformation of the TME from an ‘Immune desert’ or ‘Immune excluded’ type to an ‘Immune inflamed’ type, thereby making it more conducive for the immune system’s assault on the tumor (18, 33). Nonetheless, the precise impact of exercise on TME alterations requires further exploration. A deeper understanding of this not only sheds light on the role of exercise in cancer treatment but also may provide novel insights for the development of new immunotherapeutic strategies.

Various bioactive substances, such as myokines, endorphins, glucocorticoids, growth hormones, insulin-like growth factor-1, and nitric oxide, can be affected by exercise (34–38). These substances play a regulatory role in the immune system by modulating inflammatory responses, promoting immune cell activity, and enhancing resistance against pathogens (35, 36). Moderate and regular exercise helps maintain a balance in the levels of these substances, thereby positively regulating the immune system (10, 17, 36).

Research has shown that exercise can reduce the levels of inflammatory factors (such as TNF-α, IL-6, IL-1β) and reactive oxygen species (ROS) within tumor tissue, thus inhibiting the inflammatory immune microenvironment in tumors (22, 38). Animal studies demonstrated that aerobic exercise could reduce lung cancer inflammation, increase the levels of the anti-inflammatory cytokine IL-10, and decrease the levels of pro-inflammatory cytokines (such as TNF-α, IL-6, and IL-1) (18, 20). A systematic review and meta-analysis on lung cancer patients indicated that exercise could improve immune function, reduce inflammation, and enhance the quality of life (1). These studies suggest that exercise’s anti-lung cancer mechanism involves the regulation of tumor inflammation.

Animal and human studies indicate that exercise can affect innate immune components by elevating the levels of myeloid cells, including macrophages, monocytes, and neutrophils, in peripheral blood and tissue exudates (17, 39–41). Exercise modulates the tumor microenvironment by acting on both the innate and adaptive immune systems, increasing peripheral blood T lymphocyte and NK cell levels, enhancing their mobilization to the tumor stroma, or tumor cell cytotoxicity (18, 19, 22, 42). Furthermore, exercise can regulate the tumor microenvironment’s reprogramming by promoting myeloid cell polarization towards a more anti-tumor phenotype (15, 18, 22). However, research on exercise and the regulation of the immune microenvironment in lung cancer is limited, and the mechanism is not yet clear.

Tumor-associated macrophages (TAMs) serve a crucial role in tumor promotion (43–45). Studies have found that TAMs can constitute 30-50% of the total tumor tissue volume. Tumor cells can recruit and induce macrophages to differentiate into TAMs, which then promote tumor growth (46–48). Research has confirmed that long-term swimming training can reduce TAMs infiltration in tumor tissue and delay tumor growth (18). However, 8 weeks of voluntary wheel running does not change the number of TAMs in subcutaneously implanted leukemia tumors, suggesting that exercise’s impact on TAM accumulation in tumor tissue depends on the tumor types (18).

Endurance exercise and high-intensity interval training (HIIT) have been found not to prevent TAM accumulation in lung cancer tissue (18). Although TAMs accumulate abundantly in tumor tissue, they primarily exhibit an M2 phenotype, promoting tumor growth and immune suppression (43, 48, 49). Numerous studies are investigating how to target TAMs and polarize them from M2 to M1 phenotype to eliminate tumors (43, 47, 49, 50). Some research suggests that exercise can enhance anti-tumor activity by increasing the M1/M2 TAM ratio (18, 22). However, research indicates that endurance exercise reduces the quantity of M1-type TAMs in lung cancer tissue significantly (18). Additionally, endurance exercise downregulates TNF-α and iNOS expression in lung cancer tissue but upregulates IL-6 and IL-10 expression (18). This indicates that endurance exercise reduces the proportion of M1-type TAMs and pro-inflammatory cytokines while enhancing anti-inflammatory cytokines. HIIT also downregulates TNF-α and iNOS expression in lung cancer tissue but increases IL-12 expression and plasma IFN-γ levels. IL-12 promotes IFN-γ production, which can induce monocyte differentiation into M1-type macrophages (18). Therefore, HIIT may promote M1 polarization and immune tumor control by increasing IL-12 and IFN-γ levels. Moreover, HIIT upregulates CD47 and CD24 expression, which is associated with inhibiting macrophage phagocytic activity and enhancing tumor immunogenicity (18). In summary, HIIT may bidirectionally regulate TAM polarization and improve the immune microenvironment of lung cancer by modulating IL-10, IL-12, CD47, and CD24. In comparison, endurance exercise upregulates PD-L1 and Sirpα expression, potentially enhancing tumor immunogenicity and inhibiting macrophage phagocytic activity (18). Overall, endurance exercise and HIIT improve the immune microenvironment of lung cancer by regulating TAM polarization and immune checkpoint expression. These findings provide new therapeutic strategies for lung cancer immunotherapy.

Pedersen et al. found that exercise can mediate epinephrine-dependent and IL-6-dependent NK cell mobilization and redistribution, thus limiting tumor growth (20). In their study, gene chip analysis revealed that exercise training induced upregulation of immune function-related pathways. In tumor-bearing mice subjected to running, NK cell infiltration increased significantly, and lung tumor burden decreased (20). In addition, Asunción et al. found that aerobic and resistance training can promote myeloid tumor infiltrates (mostly neutrophils) and reduce tumor growth rate (3). These results suggest that exercise can enhance immune surveillance, increase NK cell and neutrophils infiltration into lung tumor tissue, and inhibit tumor growth.

**Figure 1:**
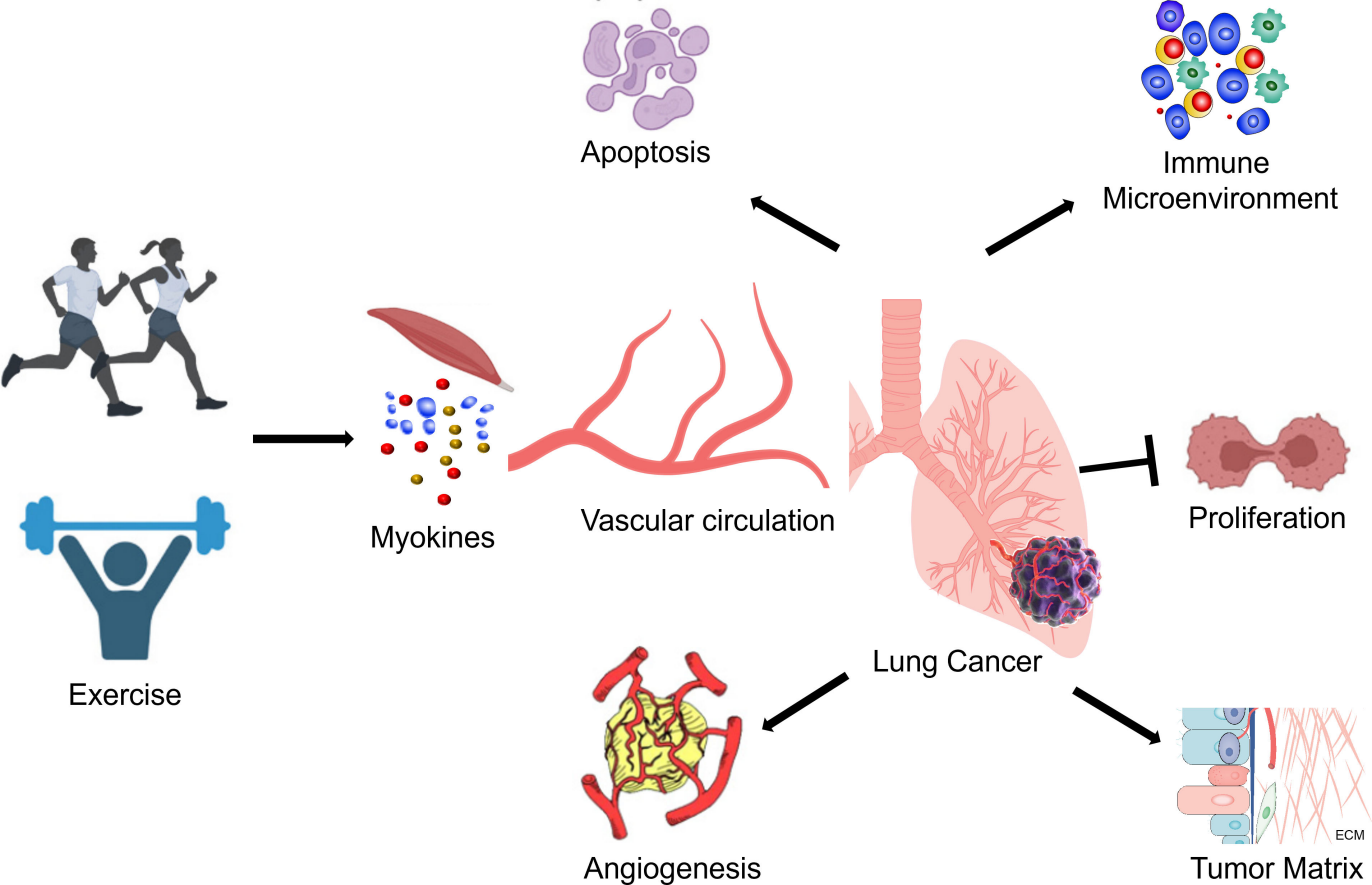
Mechanism of exercise’s anticancer influence on lung cancer.

In conclusion, exercise can influence the immune microenvironment of lung cancer tumors by modulating the production and release of bioactive substances, inflammatory factors, and immune cell activity. Exercise may regulate tumor inflammation, inhibit tumor growth, and improve the immune response by affecting the innate and adaptive immune systems, TAM polarization, and immune checkpoint expression (**Table 1**) (**Figure 1**). However, the specific mechanisms underlying these effects remain to be further elucidated.

**Table 1:** Benefits and mechanisms of exercise for lung cancer.

Species	Type	Results	Underlying mechanism	Ref
Mouse	Aerobic exercise	Slowing the progression of lung cancer	Ki67↓ MMP9↓	(23)
Mouse	HIIT	Slowing the progression of lung cancer	Ki67↓ MMP2↓	(23)
Mouse	HIIT	Diminishing the incidence of lung tumors	Unknown	(51)
Mouse	Aerobic exercise	Slowing the progression of lung cancer	p53↑; Bax↑; Ac-caspase 3↑; Apoptosis in lung cancer↑	(5)
Mouse	Voluntary Running	Showing over 60% reduction in tumor incidence and growth	NK Cell Mobilization and Redistribution	(20)
Mouse	Endurance exercise	Avoiding the resumption of tumor growth.	Protein degradation levels↓; Muscle atrophy↓	(52)
Mouse	Aerobic and resistance training	Promoting cancer immunotherapy treatment. Reducing tumor growth rate	Myeloid tumor infiltrates↑ (mostly neutrophils)	(3)
Mouse	Voluntary Running	Reducing the incidence of lung cancer	Reducing lung nodule numbers	(21)
Mouse	Aerobic exercise	Modulating the expression of some immune checkpoints in lung cancer. Reducing the proportion of M1-type TAMs in lung cancer tissues	M1 TAMs↓; SIRPα↓; PD-L1↑; Plasma IFN-γ↑	(18)
Mouse	HIIT	Modulating the expression of some immune checkpoints in lung cancer. Antagonistically regulating M1 and M2 polarization of TAMs;	IL-10, IL-12, CD47,CD24↑; Plasma IFN-γ↑	(18)

↑ means gene expression is upregulated, ↓ means gene expression is downregulated.

### The impact of exercise on the tumor microenvironment matrix in lung cancer

The effect of exercise on the matrix of the tumor microenvironment is a multifaceted process involving the extracellular matrix (ECM), matrix rigidity, matrix metalloproteinase (MMP) regulation, and modulation of extracellular signaling pathways (32, 53–55). The tumor microenvironment matrix, which includes the extracellular matrix (ECM), matrix rigidity, MMP regulation, and cell signaling modulation, plays a crucial role in tumor growth, invasion, metastasis, and treatment response (56, 57).

Exercise can affect ECM components, such as collagen and fibronectin. Some studies suggest that exercise can reduce collagen density and stiffness within the tumor microenvironment matrix, thereby slowing tumor growth and metastasis (53). However, these effects may vary depending on the tumor type and exercise modality. Tumor microenvironment matrix stiffness is closely related to tumor invasiveness and metastatic potential (58, 59). Exercise may influence ECM components and extracellular signaling pathways, altering the stiffness of the tumor microenvironment matrix. Some studies reported exercise can also affect the expression or activity of MMPs, further regulating ECM degradation and remodeling, thereby influencing tumor invasion and metastasis (22, 23). Ge et al. found that exercise can decrease MMP expression, suppressing tumor growth and metastasis (23). The study indicated that exercise can modulate the tumor microenvironment matrix composition and function by regulating extracellular signaling pathways, such as transforming growth factor-β (TGF-β), inflammatory factors, and growth factors (23). In details, high-intensity interval exercise (HIIE) can significantly increase the expression of transforming growth factor-β1 (TGF-β1) in lung cancer tissue, suggesting that HIIE may stimulate lung cancer cell epithelial-mesenchymal transition (EMT) via TGF-β1 (23). However, although TGF-β1 can activate the Smad3/4 complex and upregulate the expression of N-cadherin in non-small cell lung cancer, the impact of HIIE on N-cadherin is not well-defined, and the specific mechanism requires further investigation. Regrettably, They have shown that exercise cannot reduce the elevated expression of type I collagen in lung cancer tissue, meaning that neither mice nor HIIE can modulate lung cancer cell EMT through type I collagen regulation (23) (**Table 1**) (**Figure 1**). These findings provide new insights into the mechanisms underlying exercise’s effects on the matrix of lung cancer cells, but further research is needed to reveal more details, such as the impact of different exercise modalities on the tumor microenvironment matrix and the changes in different collagen subtypes within the matrix due to exercise.

The extracellular matrix composition is diverse and includes a range of components such as collagen, elastin, and fibronectin (54). But rare studies have reported the effects of exercise on those components. Future studies would certainly benefit from further investigation into how elastin, fibronectin, and other matrix components respond to exercise. Additionally, the influence of exercise on Cancer-Associated Fibroblasts (CAFs) within the tumor matrix warrants further exploration. CAFs are key players in tumor progression, as they contribute to matrix production, modulate immune responses, and facilitate angiogenesis (59). Thus, a deeper understanding of how exercise influences TGF-β and consequently, the behavior of CAFs, could provide key insights into the mechanism underlying the benefits of physical activity in cancer treatment. Further studies in this field could potentially pave the way for synergistic approaches combining exercise and targeted therapies.

### The impact of exercise on apoptosis in lung cancer

The influence of exercise on tumor cell apoptosis has been confirmed in numerous studies, which involve intricate mechanisms, including endocrine alterations, activation of signaling pathways, improvement of the tumor microenvironment, and regulation of apoptosis-related genes (60, 61). These findings suggest that exercise could serve as a beneficial adjuvant therapy, aiding in the suppression of tumor growth and promotion of tumor cell apoptosis.

The effect of exercise on apoptosis in lung cancer cells has been corroborated in murine models. Moderate-intensity aerobic exercise training can induce tumor cell apoptosis, activating caspases and diminishing the expression of Bcl-2 (3, 22). However, in certain models, apoptosis may not be the primary reason for tumor regression; instead, it might be associated with the response to the drug nivolumab, exercise, or their combination (62–64). Exercise can inhibit cell proliferation via multiple mechanisms, including the reduction of circulating growth factors such as insulin-like growth factor-1 (IGF-1), activation of AMP-activated protein kinase (AMPK), and inhibition of protein kinase B and mammalian target of rapamycin (mTOR) activity (5, 6).

Conversely, lung cancer tumors in exercised mice exhibit elevated levels of P53 and Bax, suggesting that exercise might enhance P53-driven apoptosis (5, 6, 18). This discovery, which involves the observation of increased functional P53 protein levels in lung cancer of exercised mice, holds significant implications for a broad range of malignant tumors. Moreover, exercise might stabilize wild-type P53 protein, rendering it more effective in tumor suppression (6). However, the current understanding of the impact of exercise on tumors carrying p53 mutations remains unclear (**Table 1**) (**Figure 1**).

Future research directions encompass the investigation of endocrine pathways, such as exercise-induced IGF-1 reduction, systemic metabolic changes, the relationship between exercise-derived exosomes and the promotion of apoptosis, and an array of related pathways (65–67).

In summary, the impact of exercise on lung cancer cell apoptosis potentially involves multiple complex mechanisms, including the activation of P53 and the modulation of endocrine pathways, thereby contributing to the deceleration of tumor growth and the promotion of tumor cell apoptosis. Further research on this topic can support that exercise can synergize with other treatment modalities (such as chemotherapy, molecular-targeting agents, immunotherapy, etc.).

### The influence of exercise on tumor angiogenesis

The impact of exercise on vascular endothelial growth factor-A (VEGF-A) remains contentious. Studies have reported decreased expression of VEGF-A in the peritumoral region or breast tumor tissue, while others have documented increased expression (68–70). Recent research indicates that after exercise intervention in mice with Lewis lung carcinoma, serum VEGF-A levels increased relative to baseline, but there were no significant disparities in survival rate or tumor growth compared to the control group (3). Alternatively, other studies have shown that high-intensity interval training (HIIT) can significantly reduce tumor VEGF-A mRNA levels, decrease tumor volume, and improve survival rates (23). But Pedersen et al. showed that voluntary running did not change expression of markers of angiogenesis (i.e., CD31 and VEGF-A) (20). In addition, exercise may lead to increased tumor blood flow (by upregulating VEGF-A), which can enhance drug delivery but may also promote tumor progression (6, 22).

Tumor growth is closely associated with the expression of Ki67 and angiogenic factors, VEGF and CD31, in tumor tissues (71, 72). CD105 is an endothelial cell marker that is highly expressed in actively proliferating endothelial cells and tumor blood vessels (73, 74). High expression of Ki67 and CD105 in tumor tissues also signifies increased tumor cell proliferation and poor prognosis (23). Ge et al. reported that both murine exercise and HIIT can reduce the expression of Ki67 and angiogenic factors in tumor tissues, suggesting that exercise may influence tumor cell proliferation by inhibiting tumor angiogenesis (23). Meanwhile, Asunción et al. has shown that moderate-intensity training can reduce the percentage of Ki67-positive cells in lung cancer tissue, suggesting that exercise can inhibit lung cancer cell proliferation (3). However, neither murine exercise nor HIIT were able to reduce the percentage of CD105-positive cells in lung cancer tissue, indicating that exercise may have a weak inhibitory effect on tumor angiogenesis (23). In addition, high expression of VEGF-C is also favorable for tumor cell metastasis. Lung cancer tissue expresses VEGF-C at substantially higher levels than normal lung tissue. However, neither murine exercise nor HIIT decreased the expression of VEGF-C in lung cancer tissue, indicating that both exercise modalities may have a modest effect on inhibiting lung cancer cell proliferation and metastasis (23) (**Table 1**) (**Figure 1**).

In summary, regular exercise can significantly inhibit local tumor growth, but its effect on suppressing distant metastasis remains uncertain and controversial. Current evidence supported that exercise may inhibit tumor proliferation by reducing the expression of Ki67 and angiogenesis-related factors in tumor tissues; however, the impact on VEGF-A/C and CD105 expression is inconsistent, indicating that exercise may have a weaker effect on inhibiting tumor angiogenesis.

### Additional mechanisms

Apart from the aforementioned mechanisms, the anticancer effects of exercise may also involve several biological processes, including autophagy, ferroptosis, anti-muscle atrophy effects and oxidative stress (52, 75–78). For example, exercise can induce cellular autophagy, a process of intracellular degradation and recycling of damaged or outdated organelles (79, 80). Exercise promotes autophagy by activating the AMPK/mTOR signaling pathway, which helps eliminate aberrant proteins and damaged cellular structures, reducing the likelihood of tumor development (81). Exercise can rescue TBI-induced ferroptosis via STING pathway (82). Exercise can also enhance the functionality of the antioxidant system, decrease reactive oxygen species (ROS) levels, and alleviate oxidative stress (83, 84). Oxidative stress is closely associated with tumor initiation and progression (85, 86). Exercise contributes to maintaining the balance between oxidative and antioxidative processes, lowering the risk of tumor development (60, 87). These mechanisms have been experimentally validated in other types of cancer, but their role in exercise as an anticancer intervention for lung cancer has not yet been confirmed. Further research is needed to explore these potential mechanisms in the context of lung cancer prevention and treatment (**Figure 1**).

## Conclusion

Exercise holds significant importance in the prevention and treatment of lung cancer. Firstly, in terms of prevention, exercise can reduce the risk of lung cancer development. By suppressing inflammatory responses, enhancing immune function, regulating cellular autophagy, and alleviating oxidative stress, exercise helps maintain a healthy physiological environment to resist cancer onset.

Secondly, in the context of treatment, exercise acts as a supplemental therapy that patients with lung cancer can benefit from in order to enhance their quality of life. Exercise helps reduce side effects during lung cancer treatment by improving cardiorespiratory function, alleviating fatigue, enhancing muscular strength, and boosting psychological well-being. Additionally, exercise can strengthen patients’ physical fitness, increasing their tolerance to other treatment modalities (such as surgery, radiotherapy, and chemotherapy), thereby enhancing treatment outcomes. Hence, we zealously promote the inclusion of exercise as a vital component in holistic lung cancer treatment plans, creating a powerhouse of support for patients. To safeguard the well-being and efficacy of exercise interventions, we propose tailoring personalized workout regimens under the expert guidance of medical professionals.

In summary, the biological basis of exercise as an anticancer intervention for lung cancer primarily includes modulation of the tumor immune microenvironment, inhibition of tumor angiogenesis, promotion of tumor cell apoptosis, and regulation of the tumor extracellular matrix. Through these mechanisms, exercise can reduce the risk of developing lung cancer, slow its progression, and enhance lung cancer patients’ quality of life. To provide a robust and comprehensive theoretical framework for clinical exercise interventions, it is imperative to engage in further investigation and unravel the intricate and nuanced anticancer mechanisms elicited by exercise within the intricate realm of lung cancer.

## Perspectives

**Figure 2:**
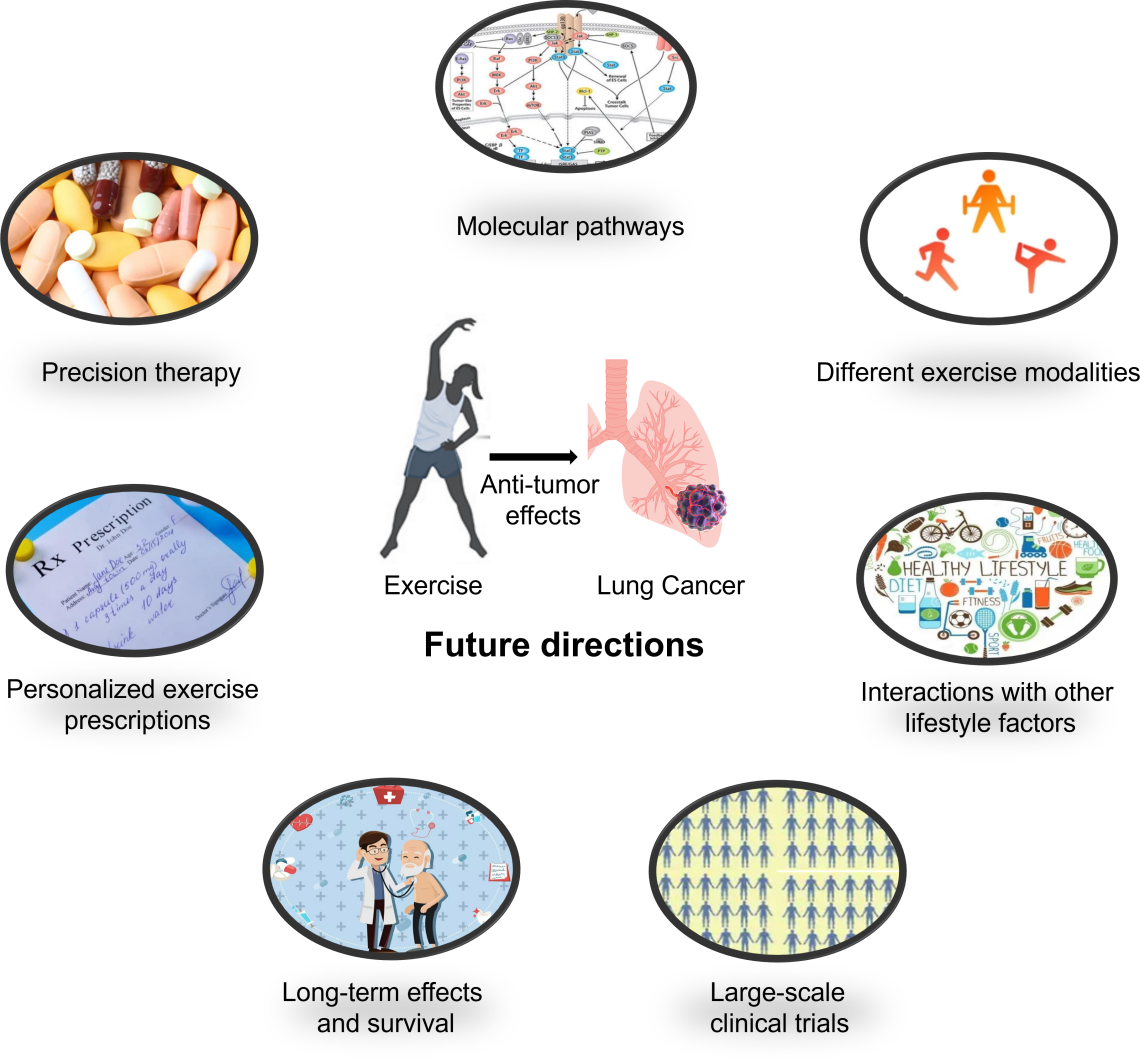
Future research directions for the treatment of lung cancer using exercise anti-tumor effects.

The fundamental research on exercise as an anticancer intervention for lung cancer will continue to delve deeper into its underlying biological mechanisms, providing more precise theoretical guidance for clinical practice (**Figure 2**). We hope that the following directions can offer insights for researchers:

1. Molecular pathways: Researchers will investigate the molecular pathways involved in the anticancer effects of exercise. This includes studying how exercise regulates the expression of genes and proteins associated with inflammation, immune responses, cell growth, and cell death in both healthy and cancerous cells (88–90).
2. Different exercise modalities: Research can also explore the optimal type, intensity, frequency, and duration of exercise interventions in animal models, providing more targeted recommendations for preventive strategies (91–93).
3. Precision therapy: Research may focus on the role of exercise interventions in combination with different therapeutic approaches, as well as the impact of exercise on specific lung cancer subtypes, disease stages, and individual patient differences, offering more personalized and precise guidance for clinical practice (92, 94–96).
4. Interactions with other lifestyle factors: Studies may investigate the interactions between exercise and other lifestyle factors (such as diet, smoking, and alcohol consumption) in terms of lung cancer risk and prognosis (87, 97). This will help develop more comprehensive lifestyle intervention strategies for more effective prevention and treatment of lung cancer.
5. Personalized exercise prescriptions: With the advancement of research, personalized exercise prescriptions may be formulated for lung cancer patients based on factors such as age, sex, physical fitness, cancer stage, and treatment plan (98, 99). These individualized prescriptions will help maximize the benefits of exercise while minimizing the risk of injuries or adverse reactions.
6. Long-term effects and survival: Future research should also focus on the long-term impact of exercise interventions on lung cancer survival, including outcomes such as overall survival, disease-free survival, and quality of life (92, 100). This will help determine the optimal duration and intensity of exercise programs for lung cancer patients and survivors.
7. Large-scale clinical trials: To further validate the benefits of exercise in lung cancer prevention and treatment, large-scale clinical trials are needed. These trials should be designed to compare the effects of different exercise modalities, frequencies, and intensities on lung cancer outcomes (94, 101).

By deepening our understanding of the mechanisms and potential applications of exercise in the context of lung cancer, we can strive to develop more targeted, effective, and evidence-based preventive and therapeutic interventions. Ultimately, this will contribute to an improvement in the general standard of life for lung cancer patients as well as a reduction in the total burden caused by this disease.

## Author contributions

SC, WQ and XS contributed to the conception and design of the study. ZL, SL and RW designed and wrote the whole manuscript. TF and ZP completed subsequent revisions of the manuscript. ZL, RW, TF and ZP collected the references and prepared figures of the manuscript. All authors contributed to the article and approved the submitted version.
